# Expert consensus on early childhood caries management

**DOI:** 10.1038/s41368-022-00186-0

**Published:** 2022-07-14

**Authors:** Jing Zou, Qin Du, Lihong Ge, Jun Wang, Xiaojing Wang, Yuqing Li, Guangtai Song, Wei Zhao, Xu Chen, Beizhan Jiang, Yufeng Mei, Yang Huang, Shuli Deng, Hongmei Zhang, Yanhong Li, Xuedong Zhou

**Affiliations:** 1 State Key Laboratory of Oral Diseases & National Clinical Research Centre for Oral Diseases & Department of Pediatric Dentistry, West China Hospital of Stomatology, Sichuan University, Chengdu, China; 2 Department of Stomatology, Sichuan Provincial People’s Hospital, University of Electronic Science and Technology of China, Chengdu, China; 3 Department of Pediatric Dentistry, Peking University School and Hospital of Stomatology, Beijing, China; 4 Ninth People’s Hospital, School of Medicine, Shanghai Jiao Tong University, Department of Pediatric Dentistry, Shanghai Key Laboratory of Stomatology, Shanghai, China; 5 State Key Laboratory of Military Stomatology, National Clinical Research Center for Oral Diseases, Shanxi Key Laboratory of Military Stomatology, Department of Pediatric Dentistry, School of Stomatology, Fourth Military Medical University, Xi’an, China; 6 State Key Laboratory of Oral Diseases & National Clinical Research Center for Oral Diseases, West China School of Stomatology, Sichuan University, Chengdu, China; 7 Department of Pediatric Dentistry, School and Hospital of Stomatology, Wuhan University, Wuhan, China; 8 Department of Pediatric Dentistry, Guanghua School of Stomatology, Guangdong Provincial Key Laboratory of Stomatology, Sun Yat‑Sen University, Guangzhou, China; 9 Department of Pediatric Dentistry, School and Hospital of Stomatology, China Medical University, Shenyang, China; 10 Department of Pediatric Dentistry, School and Hospital of Stomatology, Tongji University, Shanghai Engineering Research Center of Tooth Restoration and Regeneration, Shanghai, China; 11 Department of Pediatric Dentistry, Affiliated Stomatological Hospital, Nanjing Medical University, Nanjing, China; 12 Department of Pediatric Dentistry, Hospital of Stomatology, Jilin University, Changchun, China; 13 The Affiliated Hospital of Stomatology, School of Stomatology, Zhejiang University School of Medicine, and Key Laboratory of Oral Biomedical Research of Zhejiang Province, Hangzhou, China; 14 Department of Pediatric Dentistry, The Affiliated Stomatological Hospital of Chongqing Medical University, Chongqing Key Laboratory of Oral Diseases and Biomedical Sciences, Chongqing, China; 15 Department of Pediatric and Preventive Dentistry, The Affiliated Stomatology Hospital of Kunming Medical University, Kunming, China; 16 State Key Laboratory of Oral Diseases & National Clinical Research Centre for Oral Diseases & Department of Cariology and Endodontics, West China Hospital of Stomatology, Sichuan University, Chengdu, China

**Keywords:** Dental caries, Clinical microbiology

## Abstract

Early childhood caries (ECC) is a significant chronic disease of childhood and a rising public health burden worldwide. ECC may cause a higher risk of new caries lesions in both primary and permanent dentition, affecting lifelong oral health. The occurrence of ECC has been closely related to the core microbiome change in the oral cavity, which may be influenced by diet habits, oral health management, fluoride use, and dental manipulations. So, it is essential to improve parental oral health and awareness of health care, to establish a dental home at the early stage of childhood, and make an individualized caries management plan. Dental interventions according to the minimally invasive concept should be carried out to treat dental caries. This expert consensus mainly discusses the etiology of ECC, caries-risk assessment of children, prevention and treatment plan of ECC, aiming to achieve lifelong oral health.

## Introduction

**Fig. 1:**
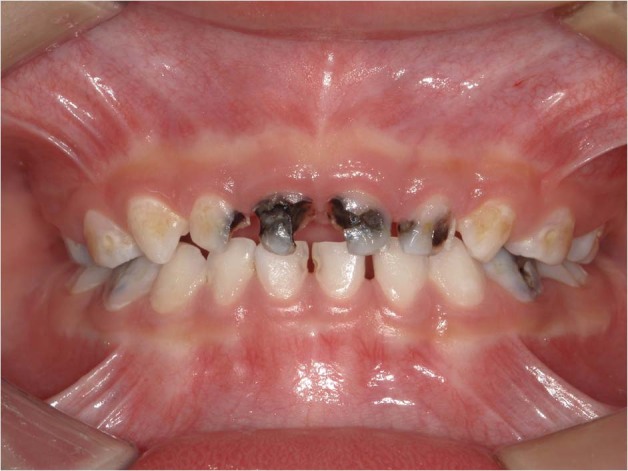
Clinical manifestation of ECC

Early childhood caries (ECC), formerly referred to as nursing bottle caries and baby bottle tooth decay remains a significant chronic disease of childhood and public health problems. ECC is defined as the presence of one or more decayed (non-cavitated or cavitated), missing (as a result of caries), or filled tooth surfaces in any primary tooth in a child 71 months of age or younger. The American Academy of Pediatric Dentistry (AAPD) also specifies that, in children younger than 3 years of age, any sign of smooth-surface caries or a dmfs (decayed, missing, or filled surfaces) score of greater than or equal to four (age 3), greater than or equal to five (age 4), or greater than or equal to six (age 5) is indicative of severe early childhood caries (S-ECC)^1^ (Fig. 1).

The experts of dental caries generally agreed that ECC was not solely associated with poor feeding practices, the term ECC better reflects its multifactorial etiology. These factors include susceptible teeth due to enamel hypoplasia, oral colonization with elevated levels of cariogenic bacteria, especially *Streptococcus Mutans* (*S.mutans*), and the metabolism of sugars by tooth-adherent bacteria to produce acid which, over time, demineralizes tooth structure^2^. Reducing the number of cariogenic microorganisms and establishing a balanced oral microenvironment will promote the remineralization of tooth and limit the disease progression. Thus, arresting caries requires behavioral modifications of the patient or caregiver and relies on the individual’s compliance in making necessary modifications^3^.

The consequences of ECC often include a higher risk of new caries lesions in both the primary and permanent dentition, hospitalizations and emergency room visits, high treatment costs, loss of school days, and diminished oral health-related quality of life.^1^

## Epidemiology of ECC

World Health Organization (WHO) mentioned that ECC is a highly prevalent global disease public health problem. The American Dental Association identified that ECC was found throughout the general child population, and was a significant public health problem in deprived communities.^4^

Firstly, the prevalence and incidence of ECC is very high, it is an early-onset, aggressive form of dental caries that affected around 1.76 billion children with primary teeth worldwide.^5^ The results of an assessment of 193 United Nations published data between 2007 and 2017 showed that the mean ECC prevalence was 23.8 and 57.3% in children younger than 3 years and children aged 3 to 6 years, respectively.^6^ The summary paper presented at the International Association of Pediatric Dentistry Conference on ECC in 2018, showed that the ECC prevalence was 17, 36, 43, 55, and 63% in children aged 1, 2, 3, 4, and 5 years, respectively.^7^ A systematic review with a sample size of 80,405 children showed the prevalence of dental caries in primary teeth was 46.2% (95% CI: 41.6%–50.8%).^8^ A systematic review using the WHO criteria, showed a combined ECC prevalence of 48%, and ECC prevalence in decades was 55% in the 1990s, 45% in the 2000s, and 49% in the 2010 decade respectively, with no significant change observed from 1990 to 2019.^9^

Secondly, although ECC is prevalent around the world, it is, in particular, growing rapidly in low- and middle-income countries.^10^ The result of the fourth National Oral Health Survey in the Mainland of China showed that the prevalence of deciduous tooth caries in 5-year-old children was 71.9%, which was 5.9% higher than that of 10 years ago, and the mean dmft (decay, missing, or filled teeth) score was 4.24.^11^ With a combined global ECC prevalence of 48%, the ECC prevalence varied both between and within countries. Ranged from 16 %(Singapore) to 89% (China), the prevalence by continent was 30% in Africa, 48% in the Americas, 52% in Asia, 43% in Europe, and 82% in Oceania, which indicated the distribution of ECC is not homogeneous.^9^ The variation could be explained by mixed factors, such as macro-economic,^12^ socioeconomic,^13^ genetic factors,^14,15^ ethnic minority populations,^16^ the availability of fluoride in drinking water^17^ or toothpaste,^18^ interventions with evidence of effectiveness for caries prevention,^19^ universal health coverage, growth of gross national income,^6^ high expenditure on health care, et al..^20^

Thirdly, the untreated primary caries remains high. ECC is still a global public health burden, medically, socially, and economically.^21^ Globally, approximately 532 million cases (95% UI, 443 to 622 million) had untreated caries in primary teeth in 2017, and between 1990 and 2017, the percentage change in the number of prevalent cases decreased in high- and upper-middle-income countries and increased in low- and lower-middle-income countries.^22^ A systematic review supported the meta-regression review showed that the prevalence of untreated caries affected 9% of children (95% CI 8.7, 9.4) in 2010 and has remained relatively unchanged for 30 years.^9,23^ Therefore, we face a huge challenge in the prevention and management of ECC.

## Research progress of ECC

ECC is a chronic infectious disease that occurs in primary teeth, which is characterized by microbiome dysbiosis with increased cariogenic bacteria. According to the fourth Chinese national oral health epidemiological survey report (2018), the prevalence of ECC in 3–5 years old children in China is ~62.5%, which is the highest chronic infectious disease in children and affects the oral and even the general health of children.

### Etiological research of ECC

The primary tooth is constricted in the cervical portion, which brings difficulty to cleaning. The primary tooth also has a lower calcium content and mineralization degree than the permanent tooth. These factors contribute to its susceptibility to dental caries. The caries microbiome plays a critical role and is the primary etiology in dental caries development. Endogenous bacteria produce weak acids as a by-product of the metabolism of fermentable carbohydrates within the formed biofilm, which causes local pH values to fall and results in the demineralization of tooth hard tissues. Therefore, the etiological study of ECC is mainly focused on oral microecological unbalance, caries core microbiome, and their relationships with host genetic factors, which contributes to the pathogenesis study of ECC and provides the theoretical basis for ecological prevention and treatment of ECC.

#### ECC core microbiome

It refers to microorganisms in dental plaque or saliva related to the occurrence of caries. Nowadays, it is well known that not only *Streptococcus* spp., *Lactobacillus* spp., and *Actinomycete* spp., but also previously unrecognized species are involved in the progression of ECC. The significant difference in microbial community structure between caries and caries-free children has been revealed, including *Veillonella* spp., *Granulicatella* spp., *Fusobacterium* spp., *Neisseria* spp., *Selenomonas* spp., and *Campylobacter* spp. Teng et al. detected that *Veillonella* spp. and *Prevotella* spp. were the main trigger of ECC instead of *S.mutans* in a 3-year cohort study.^24–26^ *Scardovia wiggsiae*, isolated from ECC, has been associated with initial carious lesions with high acid production and tolerance.^27^ A recent study found that the acquisition of the arginine deiminase system benefits *Saccharibacteria* and their host bacteria against the acidic microenvironment in the plaque biofilm.^28^ Not only bacteria but also fungi have been linked to ECC with interkingdom interactions and the abundance of *Candida albicans* is markedly higher in children with ECC than in the caries-free children.^29^

As the specific pathogen, *S. mutans* has long been the research hotspot of caries etiology. Transcription factors EpsR, StsR, RcrR, and AdcR regulate bacterial biofilm formation, sugar transportation, and zinc homeostasis.^30–33^ Gtfs acetylation modification plays an important role in biofilm formation and cariogenic virulence.^34^ As the second messengers, c-di-AMP and Ap4A are also involved in biofilm formation.^35,36^ CRISPR/Cas system regulates bacterial biofilm formation and cariogenic virulence of clinically isolated strains.^37,38^ EzrA is involved in the bacterial division, morphology maintenance, biofilm formation, and interspecies competition.^39^

Although *S. mutans* is a specific caries-associated bacterium in the initiation and progress of caries, its presence or absence is not always consistent with the severity of caries. A study explored the possible correlation of *S. mutans* and other microorganism levels on caries-concordant and discordant populations. The results found that salivary microbial communities significantly clustered based on *S. mutans* levels and independent of their caries experience. In high *S. mutans* levels groups, *Veillonella* spp., *Streptococcus* spp., and *Prevotella* spp. were significantly increased.^40^ It highlights that other species should be considered in the health/caries conditions, as well as their conjunction with *S. mutans*.

*Candida albicans* (*C. albicans*) is a Gram-positive fungal microorganism, which exists in human oral, intestinal, and vaginal mucosa.^41^ It can invade dentinal tubules and secrete acidic substances to promote enamel demineralization. It could adhere to the hydroxyapatite matrix and dissolve crystals by releasing calcium ions. *C.albicans* was detected in the oral cavity of young children with dental caries.^42^ *C.albicans* can adapt to a high acidic environment and produce high concentrations of acetic acid and pyruvate. The interaction between *C. albicans* and *S. mutans* can promote the occurrence and development of ECC.^43^

#### ECC and genetic factors

Individuals’ susceptibility to ECC is associated with genetic and environmental factors. The twins model makes it possible to identify the effect of genetic factors on oral microbial composition. The oral microbial composition of twins is more similar to each other and also showed a high similarity with their mother. In addition, there is no significant difference between the oral microbial compositions of monozygotic and dizygotic twins.^44^ These results showed that environmental factors may have a stronger effect on the composition of oral microbiota in ECC children compared with genetic factors.

### Predictors and biomarkers for ECC

In health, the oral microbiome has a symbiotic or eubiotic relationship and forms a stable dynamic balance with the host. When the balance of the oral microbiome is perturbed, it is known as “dysbiosis” and is linked to diseases. On the one hand, dysbiosis is accompanied by changes in the composition of the oral microbiome. For example, there is a higher abundance of *Corynebacterium durum* in caries-free children than in ECC children. The abundances of *Prevotella denticola*, *Megasphaera micronuciformis*, and *Dialister invisus* in ECC children are higher than in caries-free children.^45^ On the other hand, oral microbiome dysbiosis will affect the biomolecule components in saliva, as part of the host’s defense system. The identity and concentration of changing proteins are highly correlated with ECC.^46^ Thus, the changes in oral microbiota and salivary proteins in this process can be predictors and biomarkers for assessing the caries risk in children and forecasting the progress of ECC. Recently, Li et al. found that BCG-polystyrene/polyvinylpyrrolidone (BCG-PS/PVP) electrospun fibrous membrane visually detects the pH point with sensitive and fast response, which has potential application value in the monitoring and prevention of ECC.^47^

### Therapeutic strategies targeting cariogenic biofilm

The biofilm formation covering the tooth surface lays the foundation for cariogenic microorganisms to initiate the caries process. Cariogenic microorganisms live in biofilm as microcolonies that are encapsulated in an organic matrix of exopolysaccharides, protein, and DNA, which protects from desiccation, host defenses and provides resistance to antimicrobials. Consequently, biofilm formation is not interrupted and together with the absorbed saccharides from the diet leads to cariogenic microenvironments. Therefore, therapeutic strategies targeting biofilm will be effective to disrupt the pathogenic niche and prevent the progression of ECC. Multiple regulatory pathways have been demonstrated to regulate biofilm formation, including a two-component system (TCS), quorum sensing (QS) system, CRISPR/Cas system, and c-di-AMP signal system, etc.^48,49^ Inhibitors have been developed to impair the biofilm formation by targeting glucosyltransferases (Gtfs) and consequential polysaccharide synthesis: oxazole derivatives, quinoxaline derivatives, trimetrexate (TMQ), and so on.^50–54^ In addition, *Lactobacillus plantarum* K41 isolated from pickles showed high inhibitory ability against biofilm formation.^55^ With the development of dental materials, the anti-caries approaches will be further expanded. Liang et al. reported that TA@RAs, new “smart” anti-caries resin adhesives that trigger activation in response to acidic pH, showed an anti-biofilm effect and increased the microorganism’s diversity.^56^

## Management of early childhood caries

### Caries-risk assessment models for children

Caries-risk assessment (CRA) is an important part of children’s dental health care. It refers to the identification and analysis of certain factors that are considered to be related to dental caries and to propose personalized preventive and therapeutic strategies for individuals to decrease the risk of dental caries.^57^ CRA involves a comprehensive analysis of protective factors, such as fluoride use; risk factors such as the presence of caries lesions, and social, cultural factors such as social status.^58,59^ Several CRA models related to ECC have been developed worldwide, including caries-risk assessment tool (CAT), caries management by risk assessment (CAMBRA), American Dental Association (ADA) caries-risk assessment, and Cariogram (Table 1).

**Table. 1:** Factors of assessment tools for patients ≤6

	Caries-risk assessment tool (CAT) (0–5)	Caries management by risk assessment (CAMBRA) (0-6)	American Dental Association (ADA) caries-risk assessment (0–6)	Cariogram
Protective factors	Fluoride exposure; regular dental care	Fluoride exposure, Daily dental care	Fluoride exposure; dental home	fluoride program;
Risk factors	Mother or caregiver has active dental caries; poverty, low health literacy; frequent exposure to sugary snacks; frequent bottle/nonspill cup use; special health care needs; new immigrant	Frequent snacking; bottle/nonspill cup use; the family has low socioeconomic and or low health literacy status; medications that induce hyposalivation	Eligible for government programs; caries experience of mother or caregiver; special health care needs	diet contents; diet frequency;
Clinical findings	Non-cavitated caries or enamel defects; visible cavities, filling or missing teeth due to caries; visible plaque;	Plaque; decay or white spots; recent restorations	Carious lesions; Non-cavitated carious lesions; missing teeth due to caries; Orthodontic Appliances; Salivary flow	Caries experience; plaque amount; *Streptococcus Mutans*; saliva secretion; buffer capacity

CAT was developed in April 2002 by AAPD to assess caries risk in children and to aid clinical decision-making regarding diagnostic, fluoride, dietary, and restorative protocols.^60,61^ CAT is a qualitative model, that defined dental caries risk as high, moderate, and low and is mostly used for CRA in infants, children, and adolescents.^62^ CAT consists of two tables, one for children aged 0–5 and another for ≥6 years old.

CAMBRA ^31^was developed in 2002 by Calif Dent Assoc, ref. ^63^ and ref. ^64^ further completed it in 2007 and updated since then.^65,66^ CAMBRA is a qualitative model, but in the latest vision, quantitative components were added to better determine the caries-risk level.^67^ CAMBRA evaluation indicators cover risk factors, protective factors and disease indicators, and defined dental caries risk as extreme, high, moderate, and low risk. It also consists of two tables, one for 0–6 years old and another for over 6 years old (Table 2).

**Table. 2:** Caries management recommendations for patients ≤6 by CAT and CAMBRA

Category	Model	Low risk	Moderate risk	High risk	Extreme high risk
Recall	CAT	6–12 months	6 months	3 months	
	CAMBRA	6–12 months	6 months	3 months	monthly
Radiographs	CAT	12–24 months	6–12 months	6 months	
	CAMBRA	12–24 months	6–12 months	6 months	6 months
Fluoride	CAT	optimally fluoridated water/twice-daily brushing with fluoridated toothpaste	optimally fluoridated water/twice-daily brushing with fluoridated toothpaste/fluoride supplements/professional topical fluoride every 6 months	optimally fluoridated water/twice-daily brushing with fluoridated toothpaste/Professional topical fluoride treatment every 3 months/SDF on cavitated lesions	/
	CAMBRA	twice-daily brushing with fluoridated toothpaste	optimized fluoride intake/twice-daily brushing with fluoridated toothpaste/ Fluoride varnish every 6 months	optimized fluoride intake/twice-daily brushing with fluoridated toothpaste/ Fluoride varnish every 3 months	optimized fluoride intake/three times daily brushing with fluoridated toothpaste, spitting the toothpaste with no rinsing/ Fluoride varnish every 1–3 months
Dietary counseling	CAT	Yes	Yes	Yes	/
	CAMBRA	No	Yes	Yes	Yes
Sealants	CAT	Yes	Yes	Yes	/
	CAMBRA	No	On enamel defects and pits and fissures at-risk	On enamel defects and pits and fissures at-risk	All pits and fissures
Restorative	CAT	Surveillance	Active surveillance of non-cavitated /Restoration of cavitated or enlarging caries lesion	Active surveillance of non-cavitated/Restoration of cavitated or enlarging caries lesion	/
	CAMBRA	/	Active surveillance for developing lesions	Remineralize enamel-only lesion; restoration of cavitated lesions or non-surgical caries; ITR; SDF;	Caries control before surgical treatment; remineralize enamel-only lesion; restoration of cavitated lesions or non-surgical caries; ITR; SDF;
Self-managements	CAT	/	/Yes	/Yes	/
	CAMBRA	No			Yes
Additional therapies	CAT	/	/	/	/
	CAMBRA	/	/	/	use of baking soda/xylitol, ACP/CPP paste

ADA caries-risk assessment^37^ is a qualitative model, that defied dental caries risk as high, moderate, and low. The assessment has two forms, one is for patients of age 0–6, and the other is for patients over 6. The form mainly includes three aspects: (1) contributing conditions, such as fluoride exposure; sugary foods or drinks; eligibility for government programs, etc. (2) general health conditions. (3)clinical conditions, such as carious lesions, visible plaque, dental/orthodontic appliances present, salivary flow, etc.

Cariogram is a computer-based caries-risk assessment system developed by Swedish scholar Petersson et al..^68^ Cariogram is a quantitative model for caries-risk assessment. Ten caries-related factors were evaluated.^68^ By inputting seven or more indicators, a pie chart can be obtained through program operation, and the possibility of individual caries in the future can be predicted.^69^

### Management of perinatal and infant oral health

#### Oral health management for pregnant and lactating women

Changes in diet, living habits, and hormone levels during pregnancy increase the risk of dental diseases, such as gingivitis, pregnancy epulis, periodontitis, wisdom tooth periodontitis, caries, etc. These diseases not only affect the nutrition and health status of women themselves, but also impact the normal growth and development of the fetus, which are correlated with adverse outcomes in pregnancy such as prematurity, fetal growth restriction, and pre-eclampsia.^70,71^

There is a close relationship between prenatal oral health care and children's ECC. By giving prenatal oral health education or intervention, a positive ECC prevention outcome was achieved.^72,73^ When mothers were subjected to prenatal oral health promotion through education and intervention, the incidence of ECC and *S. mutans* carriage in their children may be reduced.^74^

Therefore, prenatal dental examination and education are necessary for pregnancy. Dental treatment should be carried out before pregnancy to prevent the occurrence of dental diseases during pregnancy. The dental history including diet and fluoride use, preexisting oral conditions, current oral hygiene habits, tobacco, and other substance use should be recorded.^75,76^

During pregnancy, women are advised to brush their teeth twice a day using Bass Brushing Method with fluoride toothpaste, floss daily, and visit the dentist regularly, have a balanced diet with high-quality protein, trace elements, and vitamins. Folic acid, choline, and omega-3 fatty acids are also needed.^77^ In addition, pregnant women need to be aware of diabetes, which has been correlated with congenital defects such as cleft lip and palate.^78^ Brushing should be avoided soon after vomiting in cases of morning sickness, as this practice exposes the teeth to gastric acid. For neutralization of the acid, it is recommended to rinse with a diluted solution of one cup of water and one teaspoon of baking soda.^79^ Due to the uncertain side effect, bleaching should be avoided during pregnancy.^80^ In the third trimester of pregnancy, puerperium and infant oral hygiene advice should be provided. The second trimester is the best time for dental therapy, and treatments may focus on relieving acute symptoms. All radiographic procedures should be conducted in accordance with radiation.^76^ In the third trimester of pregnancy, puerperium and infant oral hygiene advice should be provided.

#### Oral health management for the infant

Infants have underdeveloped salivary glands and less saliva secretion, also the primary teeth have a lower mineralization extent than permanent teeth, all these factors contribute to dental caries.

##### Breastfeeding

Breastfeeding is a highly effective health-promoting habit. Although breastmilk can decrease the pH value of s dental plaque and lead to its dissolution, it is less effective than infant formula.^81^ ECC may not be caused solely by breastfeeding. However, frequent feeding will elevate the cariogenic potential due to reduced salivary flow during sleep.^82,83^ Breastfeeding combined with other carbohydrates was shown to be significantly cariogenic in an in vitro study.^84^ The protective effect of breastfeeding during infancy has been revealed, which may be correlated with decreased carbohydrate intake and delay in the use of the bottle.^85^ Some studies suggested that prolonged breastfeeding for more than 12,^83^ 18,^83^ or 24 min,^86,87^ increases the caries risk, but others suggested that breastfeeding duration was not related to the high likelihood of developing dental caries, even when breastfeeding time is over 24 min.^88^ However, reducing frequent and nocturnal breastfeeding^82,89^ from the second year may decrease caries risk. The recommended duration of breastfeeding by the American Academy of Pediatrics is the first year while WHO encourages mothers to breastfeed for up to 2 years.^90^

##### Early caring

New parents may be aware of the necessity of personal oral health and the possibility of cariogenic bacteria from parent/ primary caregiver transmission to the infant. A few days after birth, daily cleaning of the infant’s gums with a clean, moist gauze pad, or a washcloth should be initiated. Brushing teeth twice daily should begin as early as when the first tooth grows. A smear or a rice-size amount of fluoride toothpaste is the standard amount that should be considered. Limit sugar consumption in meals and beverages; avert night bottle feeding with milk or sugary drinks.^91^

##### Dental visit

Infants may have their first dental examination following the growth of their first tooth, and establish a personal dental health file, which should not exceed 1 year of age at the latest(Dental Home);^92^ after that, routine dental examinations are carried out every 3 to 6 months, including dental development, Whether there are bad oral habits, caries, malocclusion, etc., and CRS is the focus.^93^ Early dental visits may also make children adapt to the medical environment and dental examination process, minimizing the occurrence of dental phobia.

### Management of caries for 0–6-year-old children

#### Fluoride

Fluoride has played a key role in decreasing dental caries, and its use for caries prevention and management is both safe and significantly effective.^94^

Low fluoride levels in plaque and saliva help remineralize demineralized enamel and prevent sound enamel demineralization. It also prevents caries via the mechanism of influencing cariogenic bacteria metabolic activity.^95^ Fluoride at high levels causes a transient calcium fluoride layer-like substance on the surface of the enamel. In the case where the value of pH was decreased due to acid formation, fluoride is liberated and can be used to remineralize enamel or affect bacterial metabolism.

The most cost-effective means to deliver fluoride to the community is through fluoridation of drinking water. In the United States, water fluoridation is carried out at the level of 0.7–1.2 mg·L^−1^. The U.S. Department of Health and Human Services has recommended standardizing all water to the 0.7 mg·L^−1^ level.^94^ In China, the optimal fluoridation level of drinking water is recommended as 0.7–1.0 mg·L^−1^.^96^

Topical fluoride therapies applied professionally are efficient in decreasing the incidence rate of dental caries. Topical fluoride therapies should be performed after completing the caries-risk assessment and by dental professionals.^94^ High-risk children should receive fluoride treatment at an interval of 3 months, and biannual treatment should be conducted for children with a moderate risk.^97^The most frequently used agents for professional fluoride treatments are 5% sodium fluoride varnish (NaF, 22 600 mg·L^−1^ F) and acidulated phosphate fluoride (APF, 12 300 mg·L^−1^ F). 5% sodium fluoride varnish in unit dosages is the only professional topical fluoride that is suitable for children under six.^98^ Clinical trials have also shown that applications for less than four minutes are noneffective. 38% silver diamine fluoride (SDF) is recommended to inhibit the progress of cavitated caries lesions in primary teeth. SDF prevents caries by acting as an antibacterial agent and by remineralization of enamel and dentin.^99^

Children’s home-use fluoride products should be used in a low-dose and higher-frequency manner.^97^ Fluoride toothpaste is indicated twice a day, and rinsing after brushing should be minimized or avoided entirely.^100^ In China, the national standard of toothpaste fluoride level is 0.05%–0.11%.^96^ For children aged less than 3 years, using an amount smaller than a smear or rice-size amount of fluoride toothpaste may reduce the incidence risk of fluorosis. Children aged 3 to 6 should use a dosage smaller than a pea-size of fluoride toothpaste.^91^ Home-use fluoride gels and pastes and prescribed strength home-use fluoride mouth rinse are efficacious as well in dental caries reduction^98^ (Table 3).

**Table. 3:** Recommended fluoride usage

Systemic use of fluoride	Professional topical use of fluoride		Home-use fluoride		
Water fluoridation	Fluoride varnish	SDF	Fluoride toothpaste^*^	Fluoride gels/pastes	Fluoride mouth rinse
0.7–1.0 mg·L^−1^	NaF (22 600 mg·L^−1^ F) ^*^	38% SDF	NaF	NaF (5 000 mg·L^−1^ F)	NaF (900 mg·L^−1^ F) (weekly)
	APF (12 300 mg·L^−1^ F)		SMFP	APF (5 000 mg·L^−1^ F)	NaF (230 mg·L^−1^ F) (daily)
			Stannous Fluoride	SnF_2_ (1 000 mg·L^−1^ F)	

*Recommended for children ≤6

#### Oral hygiene

A few days after birth, parents are advised to begin to clean infants’ gums using a clean, moistened gauze pad, or washcloth daily. Brush teeth twice a day as soon as the first tooth erupts. Brush teeth using the Bass method. Fluoride toothpaste should be used in an amount not exceeding a smear or rice-size amount.

Both the parent and the patient should be involved in oral hygiene counseling. Initially, the parent oversees the oral hygiene of the child. As the child develops, home dental care should be taken by both the parent and the child. When the child demonstrates knowledge and competence to perform personal hygiene techniques, the child should also be counseled by a healthcare professional. At each dental visit, the effectiveness of home care should be assessed.^101–103^

#### Diet habits

Diet habit is closely related to ECC, healthy diets such as lean protein and vegetable intake will promote dental health. However, unhealthy diet habits (e.g., frequent intake of sugars and/or juices) were risk factors for ECC.^104,105^ For infants, human breastmilk is recommended.^106^ But parents should be aware that breastfeeding is significantly cariogenic when combined with other carbohydrates.^84^ A healthy diet is necessary for children, including drinking plenty of water; eating various kinds of foods (whole grain, fruits, vegetables, protein, and low-fat/fat-free dairy foods); limiting the number and frequency of Sugary Snacks; balancing meals consumed with physical activity to maintain an appropriate Body Mass Index (BMI); maintaining a caloric intake adequate to sustain normal growth and development.^107^

The public and the parents should be informed about the correlation between frequent carbohydrate intake and caries, and other hazards linked to over intake of simple carbohydrates, saturated fat, and sodium.^106^

#### Pit and fissure sealant

Since the 1960s, pit and-fissure sealants have been employed^108^ to prevent and control dental caries on primary and permanent teeth. Pit and fissure sealant may protect molars from the pit and fissure lesions of occlusal surfaces, and also inhibit the growth of non-cavitated carious lesions.^109^ At 1, 2, 3, and 4 years of follow-up, studies have demonstrated that comparing resin sealant versus no sealant achieved highly significant outcomes.^108,110^

According to AAPD guidelines, it is recommended that sealants should be applied in permanent molars with both sound occlusal surfaces and non-cavitated occlusal caries in children and adolescents.^110^ Dental sealants are a cost-effective solution when antecedent caries is present. The efficiency of several types of sealants, such as resin-based sealants, resin-modified GI sealants, GI cement, and polyacid-modified resin sealants, could not be assessed owing to inadequate evidence.^108,110^

Sealing of at-risk pits and fissures should be conducted as promptly as feasible.^103^ Generally, it is recommended to have sealant for pits and fissures of primary molars at age 3–4, have sealants for pits and fissures of permanent first molars at age 6–8, and have sealants for pits and fissures of permanent second molars and premolars at age 10–12. The necessity for placing a sealant should be reevaluated at periodic prevention care sessions. Checking of sealants regularly should be conducted and fixed or changed when necessary.^110,111^

#### Dental home

The American Academy of Pediatrics proposed the concept of the medical home in 1993, from which the dental home concept is present. It is described as “the ongoing relationship between the dentist and the patient, inclusive of all aspects of oral health care delivered in a comprehensive, continuously accessible, coordinated, and family-centered way.“.^112^ It is intended to provide preventative, acute, and holistic oral health care, as well as referrals for patients, when necessary, and should be initiated as early as 6 months but not after 1 year.^113,114^ The determination of reappointment frequency is dependent on caries-risk assessment.^92,115^

Studies have well-illustrated that health-related outcomes and costs can be efficiently improved by early dental visits.^116,117^ A dental home is a useful pattern for preventing ECC. Children who lack accessibility to a dental home are exposed to a greater risk of ECC and dental treatment under general anesthesia, as shown in a Canadian study.^118^ It has been proved that conducting a dental home improves health outcomes in children, particularly those at risks of developing periodontal disease or ECC.^104^ In low-income groups, the development of a dental home reduces the incidence of ECC.^119^

### Pediatric restorative dentistry

#### Clinical techniques of caries management for toddlers

##### Atraumatic restorative technique (ART)

The University of Dar es Salaam initiated a community-based primary oral health program referred to as ART during the mid-1980s in Tanzania.^120^ It is characterized by the removal of carious tissue by only hand devices and restoring the cavity by primarily glass ionomer.

According to the WHO’s manual, ART can be used when the dentine has a cavity and it is accessible by hand equipment. It should not be employed in cases where there is swelling or fistula, the pulp is exposed, pain and inflammation symptoms develop, or the cavity cannot be accessed by hand equipment.^121^ ART is a minimal cost, physiologically friendly approach requiring little cavity preparation,^122–124^ which lowers the need of performing further endodontics and tooth extraction procedures.^125^

It has been shown that ART has a high success rate, especially for one-surface restoration. For instance, 3-year research in Zimbabwe found an 85.3% survival rate for one-surface ART restorations.^126^ Another meta-analysis has revealed that the rate of survival for single-surface ART restoration of the primary posterior teeth reached 94.3% in 2 years, and 87.1% in permanent posterior teeth in 3 years.^127^ Also, studies have found that ART may efficiently minimize pain and dental anxiety compared with traditional therapies.^128–131^

Combined use of ART and dentin conditioner/chemo-mechanical method have been developed these days. Combined use of dentin conditioner may have a better result,^132,133^ since dentin conditioner may clean the bonding surface and seal the dentinal tubules.^134^ The use of hand instruments in combination with chemo-mechanical methods will enhance the elimination of carious tissues. In addition, they may minimize pain posed by the dental treatments, making ART more suitable for children.^135,136^

##### Interim therapeutic restoration (ITR)

ITR was first developed by AAPD in 2001,^137^ it utilizes a technique similar to ART, but has different therapeutic purposes. ART was introduced in low-income nations as a treatment approach, where more appropriate treatment may not be available.^138^ ART used hand instruments only and is a definitive restoration. ITR is a temporary restoration that entails removing caries by both hands or slow-velocity rotary devices and then restoring with temporary adhesive restorative material (GIC). ITR is effective in managing dental caries of young patients, non-cooperative patients, patients with special health needs, and in situations where the conventional cavity preparation and placement are not feasible. ITR may also be used for caries control in children with multiple carious lesions before definitive restoration of the teeth.^131,139–141^ The adoption of ITR is effective in decreasing the levels of oral cariogenic bacteria promptly after its placement.^142,143^ However, if no further treatment is administered in 6 months, the bacteria counts may revert to pretreatment levels,^143^ so ITR should be changed with a more definitive restoration within 6 months. ITR is an appropriate treatment option for ECC since it slows down the deterioration of caries and enables more children to be treated.^144,145^

##### Chemo-mechanical caries removal (CMCR)

CMCR is a procedure that uses a solution to chemically soften carious tissue to facilitate its easier removal. The caries tissues were then removed by hand devices. CMCR is one of the minimal invasion caries removal techniques. This method was firstly applied in the 1970s with the aid of different reagents including ethylene diamine tetra-acetic acid (EDTA),^146^ collagenase,^147,148^ and sodium dodecyl sulfate.^147^ CMCR agents can now be grouped as enzyme-based agents and ethyl sodium hypochlorite (NaOCl).^149^

Carisolv is the best-known NaOCl-based CMCR agent. Papacarie is the commercially available Enzyme-based CMCR agent and consists of the papain enzyme, toluidine blue, chloramine, salts, a thickener, stabilizers, preservatives, and deionized water.^150^

Compared with the traditional caries removal method, CMCR showed a significant reduction of pain response and the need for local anesthesia,^151,152^ it is a useful caries removal method for anxious, disabled, and pediatric patients.^149^ However, other studies had shown that CMCR needs more clinical and technical effort and treatment time than the traditional method. This may increase fear in subjects of CMCR.^153,154^

##### Laser application

Neodymium-yttrium-aluminum-garnet (Nd: YAG) laser was the first laser innovation to be adopted in the treatment of dental problems. This laser was emerged in 1987 and got licensure in 1990 from the U.S. Food and Drug Administration.^155^

Lasers can eliminate caries efficiently with minimal disruption of adjacent tooth structure because there is a high-water content in the caries-affected tissue than in healthy tissues.^155^ The utilization of the traditional high-velocity dental equipment induces pain, discomfort, and anxiety among the pediatrics by producing vibration and noise during dental restorative procedures.^156–158^ Erbium lasers are contactless with the hard tissue and do not generate vibrations like the handpiece devices.^156–158^ Therefore, they have been discovered to exert a pain-relieving effect on the target hard tissues, minimizing the usage of anesthesia and injections during tooth preparations.^159–161^

#### Restoration

##### Preventive resin restoration (PRR)

PRR is a restorative method first proposed by ref. ^162^ in 1978 for the management of pits and fissures that have minimal or questionable caries. Indications for PRR are questionable caries, or an explorer caught in a pit or fissure; minimal, shallow pit and fissure caries; deep pits and fissures that could inhibit complete penetration of sealant material or could be carious at their bases; deep pits and fissures with obvious supplemental fissuring and limited areas of decay; and an opaque, chalky appearance along with pits and fissures that could indicate early-stage caries. PRR is contraindicated for large, deep, or multisurface carious lesions.^163–165^ It is characterized by only removing a small number of teeth, repairing early carious lesions, and protecting the unprepared area from secondary caries. Classic PRR were prepared with a small round bur and the procedure was completed with composite resin and pit and fissure sealant. The success rate of PRR is high, even after a long period of time.^166,167^ Nowadays, PPR has been combined with other technologies, such as Laser application to produce better long-term results.

##### Resin-based composite (RBC) restoration

As an essential component in pediatric restorative dentistry, RBCs are composed of chemically bonded fillers and a resin matrix^168^ and are classified according to the size of their fillers, since the fillers influence the esthetics/polish ability, polymerization shrinkage, depth, and physical features. Hybrid resins involve different particle sizes to enhance strength while retaining esthetics.^169^ The larger filler particles enhance strength whereas the smaller particles improve esthetics/polish ability. In comparison with hybrid resins, flowable resins have a smaller volumetric filler percentage.^170^ RBC may be utilized in PRR, Class II restorations in both the primary^171,172^ and permanent dentition,^173–175^ and indirect RBC restorations in both the primary and permanent dentition.^176,177^

Operator expertize, restoration size, and tooth position are all factors that have the potential to promote the durability of resin composites.^71^ Resins are more sensitive to the technique in contrast to amalgams. If the patient is uncooperative or there is an isolation problem, the usage of RBC may not be the best choice.^178,179^ Before the operation, a caries-risk assessment must be taken, and children at high caries risk are also not good candidates for RBC.

##### Glass-ionomer cement (GIC) restoration

GIC have been used in dentistry as restorative materials since the early 1970s.^180^ GIC have several features that enhance its usage among pediatrics: the ability to chemically bond to both dentin and enamel, biocompatibility, uptake and release of fluoride lower moisture sensitivity than resins, and relative thermal expansion ability to teeth structures.^181^ However, they have poor resistance to wear, unfavorable mechanical properties as well as poor esthetics.^182^

Fluoride is released from the GIC and taken up by the surrounding dental structures.^183^ Studies have shown that conventional GIC is suitable for Class I restorations in primary molar,^184^ as conventional GIC has a median failure time of 1.2 years,^185^ and has poor anatomical form and marginal integrity.^186^

Resin-modified glass-ionomer cements (RMGICs) contain hydroxyethyl methacrylate (HEMA) and can be light cured.^187^ It has better mechanical qualities^188^ and improved resistance to moisture contamination compared to traditional GIC, while the fluoride release stays constant.^189^ RMGICs are efficient in primary teeth and can be used for both Class I and Class II restorations in the primary dentition.

GICs are also used in ART and ITR technologies and are a suitable base or liner when RBC is used as the restorative material.

##### Giomer restoration

Giomer is a new type of hybrid material that was first introduced in the early 2000s,^190^ it is made up of pre-reacted glass ionomer (PRG) filler particles within a resin matrix.^191^ PRG filler is generated by an acid-base reaction between fluoride-containing glass particles (fluoro-boro-aluminosilicate glass filler) and polyalkenoic acid with water before integration into the resin.^192^

Giomer has the features of both glass ionomers and resin composites such as the capacity of releasing and recharging fluoride, biocompatibility, and good esthetics.

In vivo clinical studies reveal that the morphological, mechanical integrity, and functional properties of the giomer restorations can be compared favorably with resin composite restoratives.^193,194^ Studies have also shown that Giomer has a high success restoration rate. A clinical trial used Giomer as Class I and Class II restoration in a permanent molar and showed that the 8-year success rate reached 100%,^192^ and the 13-year success rate is still pretty high.^195^

##### Stainless steel crown (SSC) for primary molar

**Fig. 2:**
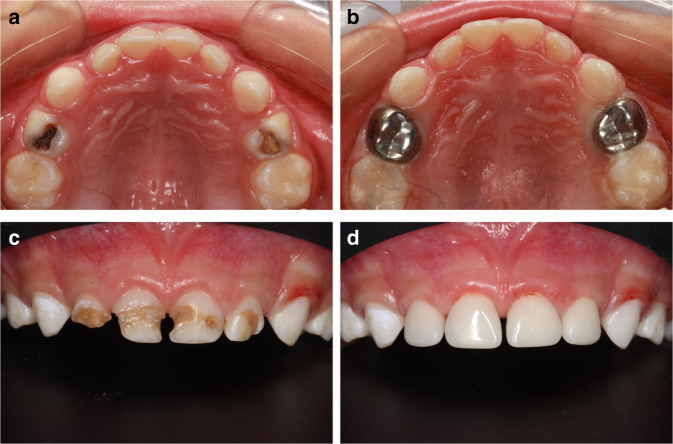
Application of Stainless steel crown and Anterior esthetic restoration, **a** Primary molars with multisurface caries, **b** Primary molars restored with Stainless steel crown restoration, **c** Primary incisors with multisurface caries, **d** Primary incisors restored with strip crowns

SCC are prefabricated crown forms that are adjustable to a patient’s tooth and cemented with a biocompatible luting agent. SCC has shown greater longevity than amalgam^196^ and RMGIC^197^ restorations. SSCs continue to offer the advantage of full coverage in the fight against recurrent dental caries and provide strength and extended durability with minimal maintenance requirements, which are favorable for high-risk pediatrics.^181^ Evidence from retrospective research studies shows that preformed metal crown restorations last longer in comparison to resin-based restorations or amalgam in caries in primary teeth treatment. Therefore, the use of SSCs on high-risk pediatric with extensive or multisurface cavitated or non-cavitated lesions on deciduous molars is recommended, particularly in the case when children need behavioral guidance approaches advancements, such as general anesthesia to provide restorative dental care^181^(Fig. 2).

Hall Technique (HT) was first developed by a general dental practitioner, Dr. Norna Hall in 1997.^198^ HT is a noninvasive treatment, applying SCC to a caries primary molar by separating teeth rather than removing caries tissues and preparing the tooth, so HT may need no local anesthesia, caries removal, or tooth preparation.^196^ HT avoids the discomfort from local anesthesia and caries removal provides a treatment option for anxious children.^199^ Also, avoiding caries removal may prevent pulpal exposure.^200,201^ When HT is used, decay is sealed under SSC, denying biofilm microbes their source of nutrition, dietary carbohydrate, and removing this access prevents the progression of caries.^202^ Studies have shown that HT had a similar success rate with conventional crowns^203^ and has a favorable restoration longevity.^201,204^ Another RCT study compared the 36-month outcome of HT and ATR showed that HT had almost three times higher survival rates for restoring primary molars than ART^130^ Both HT and ART are well-accepted among children and their parents.^205^ Therefore, HT may be used when conventional crowns are not feasible.^181^

##### Anterior esthetic restoration in primary incisors

Anterior restoration of primary incisors can be cumbersome in pediatrics due to small teeth size, minimal surface area for bonding, proximity between teeth pulp and surface, and the child’s behaviors.^206^

The following are indications for full coronal restoration of carious primary incisors: caries is extensive on the surfaces; involvement of the incisal edge; extensive cervical decalcification; pulpal therapy is recommended; the presence of minor caries with poor oral hygiene; or unfavorable child’s behavior that hinders moisture control.^207^

Retrospective research has revealed that at least 80% of strip crowns were completely retained after 3 years while those that were partially retained accounted for 20%, with none being lost.^208^ Another retrospective study showed 80% retention of strip crowns after 24–74 months.^209^ For full coronal coverage restorations in primary anterior teeth, strip crowns are a treatment option (Fig. 2).

### Deep caries and vital pulp therapy

#### Indirect pulp therapy (IPT)

IPT is a procedure that leaves the deepest caries adjacent to the pulp undisturbed to avoid pulp exposure. Then, a biocompatible material is used to cover caries-affected dentin to provide a biological seal.^210^ A dentin bonding agent like calcium hydroxide, resin-modified glass ionomer, or MTA is usually used on the remaining carious dentin, to trigger dentin’s repair and healing process. The tooth then is restored with a dental material. IPT is indicated in primary teeth with deep caries that exhibit no pulpitis or with reversible pulpitis when the deepest carious dentin is not removed to avoid pulp exposure. James A. Coll et al. reviewed articles and found that the success rate for IPT was 94.4% at 24 months, and liner material (Calcium hydroxide liners versus bonding agent liners) did not affect the IPT success.^211^ Successful IPT is possible under defined conditions (symptom-free tooth, no pulp exposure) and appropriate sealing of the cavity with an effective dentine seal.

#### Pulpotomy

Pulpotomy is performed in a primary tooth when caries removal results in a pulp exposure in a tooth with a healthy pulp or reversible pulpitis and there is no radiographic sign of infection or pathologic resorption. The coronal pulp is amputated, pulpal hemorrhage controlled, and the remaining vital radicular pulp tissue surface is treated with a long-term clinically-successful medicament.^212^ The meta-analysis showed that the 2-year overall success rate of pulpotomy was 82.6%.^211^ MTA is the only recommended medication for teeth to be retained for 2 years or longer.^210^ Then the tooth is restored with a restoration material (such as glass-ionomer cement, resin-based composite, giomer, or stainless steel if necessary) to prevent the tooth from microleakage.

**Fig. 3:**
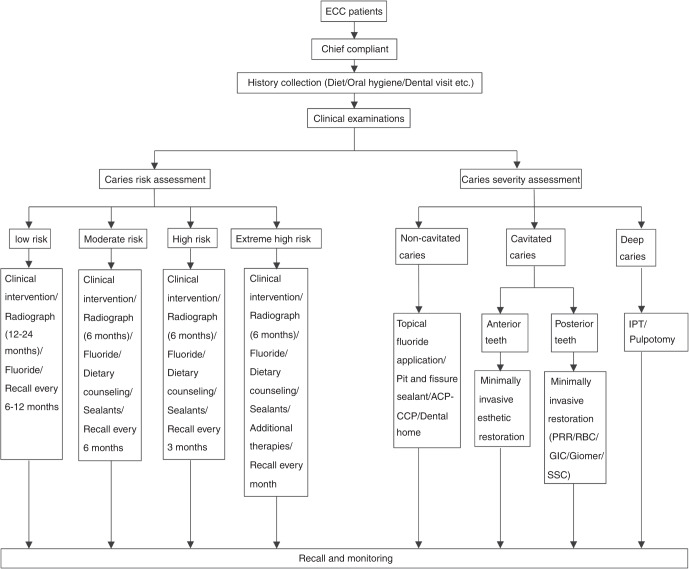
Management decision model of ECC

Management of ECC begins at the mother’s pregnancy. Early establishment of healthy dietary habits, oral hygiene habits, and dental home is essential to children’s dental health. An individualized ECC management plan should be made based on the assessments of caries risk and the clinical evaluation of caries lesions (Fig. 3).

## Assessment and management after treatment for ECC

Dental caries intervention is insufficient alone in halting the progression of the disease.^62^ Therefore, after treatment for ECC, continuous assessment and management of the health status of the patient should be performed to manage oral health in the long term.

CRA is a key component of current preventive care for infants, children, and adolescents. It should begin as soon as the first primary tooth emerges and be reevaluated regularly by dental and medical practitioners,^62,213^ and 12‐month anticipation is more accurate than a long-term duration.^7^

### Secondary caries

Secondary caries is the most common cause for the replacement of dental restorations in clinical settings, no matter what kind of material is chosen, secondary caries cannot be completely avoided.^214^ A number of factors may be responsible for secondary caries: (1) clinical technique,^214^ moisture control, visual inspection, and for children, behavioral management, etc. are all clinical factors that may predispose the development of secondary caries. (2) microleakage at the tooth-restoration interface.^215^ Until now, no material can completely eliminate microleakage around the restoration. Immediately after the use of adhesives, a gap of 6–10 μm is formed between the tooth tissue and the restoration.^216^ Furthermore, the level of microleakage was not affected by conventional or chemical-mechanical methods of caries removal,^217^ (3) microbiological change of restoration area,^218^ (4) restoration material properties,^214^ Fluoride-releasing restorative materials such as GIC or giomer may have advantages over resin-based materials,^214^ (5) oral hygiene is also closely related to the occurrence of secondary caries.^219^ The prevention methods of secondary caries include using fluoride-releasing materials; microleakage control, adequate plaque removal, especially the gingival part of the restoration by toothbrushing and interdental flossing, and adequate fluoride contact will help prevent secondary caries.

### Ecological balance

The human and symbiotic microbes form a complex ecosystem whose dynamic balance is significantly correlated with physical health. Frequent dietary carbohydrate consumption may result in dysbiosis of the oral microbial community from an overproduction of acid with selection for elevations in acidogenic, acid-tolerant bacteria.^220,221^ For example, *S.mutans*, *Scardovia wiggsiae, Slackia exigua, Granulicatella elegans*, and *Firmicutes* were found to be predominant in the plaque biofilms of carious lesion. In contrast, bacteria such as *Streptococcus cristatus, S. gordonii, S. sanguinis, Corynebacterium matruchotii*, and *Neisseria flavescens* were common in plaque biofilm of noncarious, healthy, tooth surfaces.^222^ Therefore, rebalancing the caries microbiome dysbiosis after ECC treatment is of importance.

Timely dental caries restoration, mechanical removal of dental plaque, the use of antimicrobial compounds, diet modification, and topical fluoride application may help with oral ecological balance.^223^ As sugar intake influences microbiome dysbiosis, sugar substitutes such as Xylitol and erythritol are encouraged to prevent cariogenic bacteria transmission.^224^

## Conclusion and expectation

Until now, ECC is still a worldwide health challenge. The caries microbiome plays a critical role in the occurrence of ECC. Based on the etiological research of ECC, new biomarkers and therapeutic strategies may be developed for the better prevention and treatment of ECC. Maternal oral health and awareness of health care are directly related to the oral health of infants and young children. Oral health promotion, including education and healthcare services during pregnancy, is necessary to improve infants' and young children’s oral health. A dental home should be established at an early stage of childhood and an individualized caries management plan should be provided according to caries-risk assessment. Active measurements adhering to the concept of minimum intervention should be taken to treat dental caries. In China, the prevalence of ECC is still increasing, whole-life-cycle caries management should start at the very beginning of one’s life to effectively prevent and treat caries to achieve the aim of lifelong oral health.
